# Using causal interventionist models to examine the relationship between social anxiety and paranoia: A 3-month follow-up cross-cultural survey conducted in thailand and the united kingdom

**DOI:** 10.1192/j.eurpsy.2021.1379

**Published:** 2021-08-13

**Authors:** W. Aunjitsakul, H. Mcleod, A. Gumley

**Affiliations:** Institute Of Health And Well-being, Medical, Veterinary and Life Sciences College, University of Glasgow, Glasgow, United Kingdom

**Keywords:** Shame, Prospective Studies, Psychotic disorders, Cross-Cultural Comparison

## Abstract

**Introduction:**

The continuum of social threat ranges from anxiety to paranoia. Examining the factors that predict and mediate the relationship between social anxiety and persecutory paranoia will help with the development of interventionist-causal theories that can guide the development of new treatments.

**Objectives:**

To investigate mediators between social anxiety and persecutory paranoia in a prospective cross-cultural analogue sample.

**Methods:**

A 3-month follow-up online survey included participants aged ≥18-years-old in Thailand and the UK. Recruitment was via advertisements on websites and social media. Participants completed questionnaires at baseline (T1) and 3-month follow-up (T2) measuring social anxiety, paranoia, depression. Mediators were: stigma; internal and external shame; social rank; self-esteem; and safety behaviours. We used linear regression to examine predictors of paranoia and mediation analysis to test indirect effects. Estimating the indirect effects was calculated by 10,000 bootstrapping bias-corrected 95% confidence intervals.

**Results:**

At follow-up, 186 (70.4%female; mean age 34.9±9.1) Thai and 236 (81.4%female; 35.7±12.7) UK respondents completed the survey. Regarding change scores (T2-T1), higher paranoia was significantly predicted by higher social anxiety and external shame controlling for age, gender, depression. A simple mediation model controlling for depression showed significant indirect effects for external shame (ab=0.06, 95%CI=0.018 to 0.105) and safety behaviours (ab=0.06, 95%CI=0.002 to 0.127). A multiple mediation model found external shame was a significant mediator (ab=0.06, 95%CI=0.020 to 0.110).

**Conclusions:**

These cross-cultural data suggest that external shame may mediate the prospective relationship between social anxiety and paranoia. These data suggest the potential for treatment of persecutory fears and social anxiety in psychosis by targeting shame-related cognitions.

